# Critical evaluation of indirect methods for the determination of deoxynivalenol and its conjugated forms in cereals

**DOI:** 10.1007/s00216-015-8793-0

**Published:** 2015-06-12

**Authors:** Alexandra Malachová, Lenka Štočková, Astrid Wakker, Elisabeth Varga, Rudolf Krska, Herbert Michlmayr, Gerhard Adam, Franz Berthiller

**Affiliations:** Christian Doppler Laboratory for Mycotoxin Metabolism and Center for Analytical Chemistry, Department of Agrobiotechnology (IFA-Tulln), University of Natural Resources and Life Sciences, Vienna (BOKU), Konrad Lorenz Str. 20, 3430 Tulln, Austria; Department of Crop Science, Breeding and Plant Medicine, Faculty of Agronomy, Mendel University in Brno, Zemědělská 1, 61300 Brno, Czech Republic; Crop Research Institute, Drnovská 507/73, 16106 Prague 6-Ruzyně, Czech Republic; Laboratory of Food Analysis, Department of Bioanalysis, Ghent University, Harelbekestraat 72, 9000 Ghent, Belgium; Department of Applied Genetics and Cell Biology, BOKU, Konrad Lorenz Str. 24, 3430 Tulln, Austria

**Keywords:** Masked mycotoxins, LC-MS/MS, Chemical hydrolysis, Wheat, Barley, Maize

## Abstract

A critical assessment of three previously published indirect methods based on acidic hydrolysis using superacids for the determination of “free” and “total” deoxynivalenol (DON) was carried out. The modified mycotoxins DON-3-glucoside (D3G), 3-acetyl-DON (3ADON), and 15-acetyl-DON (15ADON) were chosen as model analytes. The initial experiments focused on the stability/degradation of DON under hydrolytic conditions and the ability to release DON from the modified forms. Acidic conditions that were capable of cleaving D3G, 3ADON, and 15ADON to DON were not found, raising doubts over the efficacy of previously published indirect methods for total DON determination. Validation of these indirect methods for wheat, maize, and barley using UHPLC-MS/MS was performed in order to test the accuracy of the generated results. Validation data for DON, D3G, 3ADON, and 15ADON in nonhydrolyzed and hydrolyzed matrices were obtained. Under the tested conditions, DON was not released from D3G, 3ADON, or 15ADON after hydrolysis and thus none of the published methods were able to cleave the modified forms of DON. In addition to acids, alkaline hydrolysis with KOH for an extended time and at elevated temperatures was also tested. 3ADON and 15ADON were cleaved under the alkaline pH caused by the addition of KOH or aqueous K_2_CO_3_ to “neutralize” the acidic sample extracts in the published studies. The published additional DON increase after hydrolysis may have been caused by huge differences in matrix effects and the recovery of DON in nonhydrolyzed and hydrolyzed matrices as well as by the alkaline cleavage of 3ADON or 15ADON after the neutralization of hydrolyzed extracts.

## Introduction

Deoxynivalenol (DON) is the most frequently occurring mycotoxin worldwide, particularly in cereal crops such as wheat, maize, barley, oats, and rye, and less often in rice, sorghum, and triticale [1]. It belongs to the trichothecenes, a family of closely related compounds produced mainly by *Fusarium* spp. [1]. *F. graminearum* and *F. culmorum* are responsible for* Fusarium* head blight in wheat (scab) and* Fusarium* ear rot in maize [2], and are considered to be the most important producers of DON. A direct relationship between the incidence of* Fusarium* head blight and the contamination of wheat with DON has been established [3].

Plants have a versatile detoxification system that can deal with a wide range of xenobiotics. As DON interacts with vital cell functions of infected plants, it also represents a target for plant defence systems. Basically, detoxification mechanisms involve three major phases, consisting of chemical modification (phase I and II metabolism) and compartmentation (phase III metabolism), resulting in the formation of so-called masked mycotoxins [4]. This term was introduced by Gareis et al. [5], and refers exclusively to plant metabolites [4]. Chemical modifications are achieved by linking polar moieties to the parent toxin, leading to a decrease in toxicity towards plants. The modified toxins are partly stored in vacuoles (extractable forms) or irreversibly bound to macromolecules (nonextractable forms) [4]. The major pathway in the biotransformation of DON is conjunction with a glucose moiety, forming deoxynivalenol-3-β-D-glucoside (D3G) [6]. Ever since an analytical standard of D3G first became commercially available, D3G has been reported as a co-contaminant with DON of cereals and cereal-based products, as reviewed by Berthiller et al. [4]. Moreover, increasing D3G levels have been observed during malting and brewing [7]. In addition, DON-diglucoside and oligoglycosylated DON conjugates with up to four bound hexose units were identified in malt and beer using several sample preconcentration steps prior to analysis performed by liquid chromatography–high-resolution mass spectrometry (LC-HRMS) [8]. Only recently, several other masked forms of DON (DON-*S*-cysteine, DON-*S*-cysteinyl-glycine, DON-glutathione) have been identified in wheat using an innovative strategy of untargeted screening based on stable isotope labeling followed by LC-HRMS measurements [9].

However, the formation of mycotoxin conjugates is not limited to plant defence mechanisms. For instance, the acetylated forms 3-acetyl-deoxynivalenol (3ADON) and 15-acetyl-deoxynivalenol (15ADON) are fungal precursors of DON, although they can also occur as intermediates during DON detoxification in plants [10]. Moreover, mycotoxins can also be modified by bacteria and mammals [4]. Thus, the term “modified mycotoxins” has been introduced to cover all structurally altered forms of mycotoxins, independent of their origin [11]. D3G and ADON can potentially be hydrolyzed to DON during food processing or in the digestive tracts of mammals, and thus contribute to the total dietary exposure to DON [8].

**Fig. 1:**
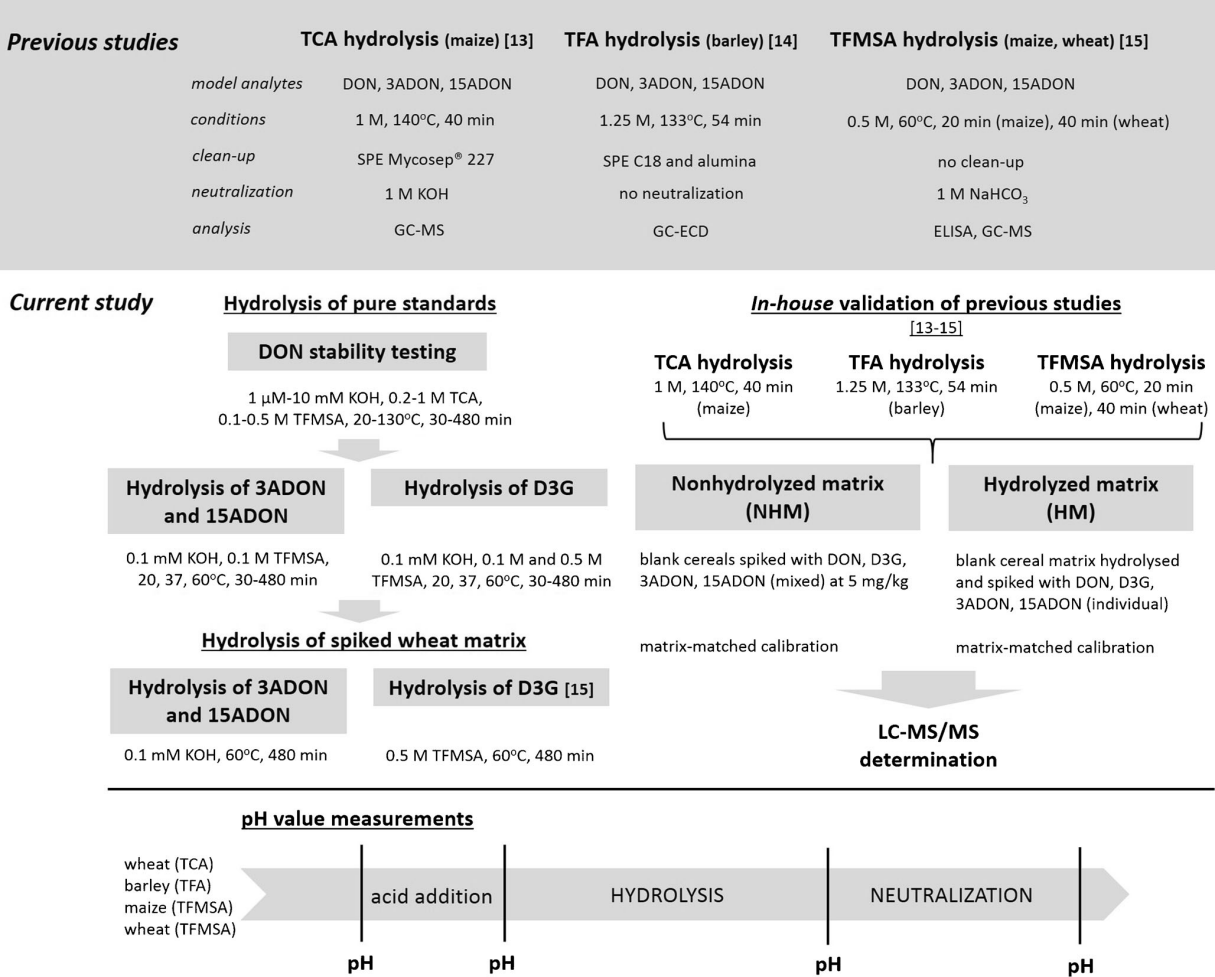
Overview of three published chemical hydrolysis methods [13–15] for total deoxynivalenol (DON) determination in cereals, and the scheme of the experiments conducted in this study.* 3ADON* 3-acetyl-deoxynivalenol,* 15ADON* 15-acetyl-deoxynivalenol,* D3G* deoxynivalenol-3-glucoside,* TCA* trichloroacetic acid,* TFA* trifluoroacetic acid,* TFMSA* trifluoromethanesulfonic acid

Little is known about the toxicological relevance of modified mycotoxins, but data from the literature show that conjugates of xenobiotics can be toxicologically significant. The Panel on Contaminants in the Food Chain of the European Food Safety Authority decided on a pragmatic approach to human risk assessment by assuming that all modified mycotoxins have the same toxicities as their parent compounds [12]. In line with that, the use of an indirect approach to determine mycotoxins in food and feed seems to be an attractive alternative to target analysis. Indirect methods aim to determine the entire “pool” of modified mycotoxins (extractable and nonextractable forms) in a sample by converting them into the parent toxin using chemical or enzymatic hydrolysis. The main advantages of this approach are that chemical standards of the modified forms are not required and as-yet undetected compounds can also be accounted for. A few indirect methods for the determination of modified forms have also been developed, as reviewed by Berthiller et al. [4]. Three of them deal with DON determination based on chemical hydrolysis (an overview is provided in Fig. 1) [13–15]. Liu et al. published an indirect approach based on trichloroacetic acid (TCA) hydrolysis followed by gas chromatography coupled with electron capture detection (GC-ECD) [13]. Similarly, trifluoroacetic acid (TFA) and trifluoromethanesulfonic acid (TFMSA) hydrolysis were used prior to total DON determination in barley [14] and in maize and wheat [15], respectively. GC-MS as well as GC-MS together with enzyme-linked immunosorbent assay (ELISA) were applied in the latter two studies.

The chemical hydrolysis of carboxylic acid esters is a pH-dependent process and is possible under either acidic or alkaline conditions. Acidic hydrolysis of esters involves initial protonation of the carbonyl oxygen. The polarization of the carbonyl group shifts some of the electron density away from the carbon atom, making it more electrophilic and therefore susceptible to the nucleophilic addition of water. Alkaline hydrolysis of esters proceeds via direct nucleophilic addition of the hydroxide ion to the carbonyl group, as it is a stronger nucleophile than water [16]. Glycosidic bonds can also be cleaved chemically. While glycosides are generally susceptible to acidic conditions, in some cases they are susceptible to basic conditions too. This sensitivity to acids is attributed to the sugar moiety, while the nature of the aglycon is more responsible for the instability of glycosides under basic conditions [17].

The aim of the study reported in the present paper was to provide a critical assessment of indirect methods for determining total DON based on acidic hydrolysis. The stability/degradation of DON, D3G, 3ADON, and 15ADON was tested using spiked samples of wheat, maize, and barley and by employing a liquid chromatographic tandem mass spectrometric (LC-MS/MS) method which was developed and validated for this purpose. The workflow of the experiments performed is also shown in Fig. 1.

## Experimental

### Chemicals and reagents

Methanol, acetonitrile (both LC gradient grade), glacial acetic acid (p.a.), potassium hydroxide (p.a.), and anhydrous sodium bicarbonate were purchased from VWR International GmbH (Vienna, Austria). Anhydrous potassium carbonate (p.a., ≥99 %) was obtained from Fluka Chemie AG (Buchs, Switzerland). Ammonium acetate (MS grade), trifluoroacetic acid (reagentplus^®^, 99 %), trichloroacetic acid (ACS reagent, ≥99 %), and trifluoromethanesulfonic acid (reagent grade, 98 %) were obtained from Sigma–Aldrich (Vienna, Austria). Hydrochloric acid (fuming, 37 %, p.a.) was purchased from Merck KGaA (Darmstadt, Germany). Water was purified using a Purelab Ultra system (ELGA LabWater, Celle, Germany). Buffer solutions for calibrating the pH meter, disodium hydrogen phosphate/potassium hydrogen phosphate (pH 7.00 ± 0.01, 20 °C), boric acid/potassium chloride/sodium hydroxide (pH 10.00 ± 0.02, 20 °C), and citric acid/sodium hydroxide/hydrogen chloride (pH 4.00 ± 0.01, 20 °C) were purchased from Merck KGaA. Standards for DON, 3ADON, and 15ADON were purchased from Romer Labs GmbH (Tulln, Austria) as stock solutions of 100.8 μg mL^−1^, 100.1 μg mL^−1^, and 103.3 μg mL^−1^, respectively, in acetonitrile. D3G was initially purified from wheat plants treated with DON at anthesis [18] or prepared by enzymatic synthesis using a purified recombinant UDP-glucosyltransferase (Michlmayr et al., in preparation). A 200 μg mL^−1^ stock solution in methanol was used to prepare the standards. The concentration of D3G was verified with a liquid calibrant of D3G (purity > 95 %) in acetonitrile obtained from Romer Labs.

### Samples

Ground blank wheat, barley, and maize samples were used as model matrices for the spiking experiments. Wheat and maize were grown by Prof. Marc Lemmens at the Institute for Biotechnology in Plant Production (IFA-Tulln, BOKU, Austria) and a barley sample was kindly provided by Assoc. Prof. Radim Cerkal (Mendel University, Brno, Czech Republic). The artificially inoculated wheat sample of the variety “Sultan” used in this study was provided by the Crop Research Institute (Prague, Czech Republic).

### Hydrolysis experiments with pure standards

The stability of DON was tested under various hydrolytic conditions. For each of the tested conditions, 35 μL of DON stock solution (100.8 μg mL^−1^) were evaporated to dryness under a gentle nitrogen stream in triplicate and the residue was re-dissolved in 7 mL of the hydrolysis agent. Aqueous solutions of KOH (1 μM, 0.1 mM, 0.1 M), TCA (0.2 M, 0.5 M, 1 M), and TFMSA (0.1 M, 0.2 M, 0.5 M) were used for 30, 70, 120, 240, and 480 min at 20 °C, 37 °C, 60 °C, 100 °C, and 130 °C. TFA was not used in these initial experiments because it was not in stock. The following temperatures were maintained: (i) 20 °C (room temperature), (ii) 37 °C and 130 °C using a Kelvitron T type T6120 laboratory oven (Heraeus Instruments, Hanau, Germany), and (iii) 60 °C and 100 °C using an IKAMAG RET-G laboratory heater (IKA Labortechnik, Staufen, Germany). Afterwards, the hydrolyzed mixtures were neutralized with stoichiometrically equivalent amounts of HCl or KOH and measured with UHPLC-MS/MS. The neutrality of each solution was confirmed by pH measurements.

The conditions under which DON was found to be stable were used for further hydrolysis experiments with D3G, 3ADON, and 15ADON. Similarly, 1 μg of single dried-down standards of D3G, 3ADON, and 15ADON were prepared in triplicate and hydrolyzed with 4 mL of (i) D3G: 0.1 mM KOH, 0.1 M TFMSA, 0.5 M TFMSA at 20, 37, and 60 °C for 30, 70, 120, 180, 240, and 480 min; (ii) 3ADON and 15ADON: 0.1 mM KOH, 0.1 M TFMSA at 20, 37, and 60 °C for 30, 60, 120, 180, 240, 480 min as well as 16, 20, and 24 h. Neutralization after hydrolysis was performed with appropriate amounts of HCl or KOH. Again, neutrality was confirmed via pH measurements. Samples were subsequently measured with UHPLC-MS/MS.

### Hydrolysis of conjugated DON in the wheat matrix

As naturally contaminated wheat samples containing only 15ADON or 3ADON (without large amounts of D3G) were not available, the experiments were performed with spiked samples. Both a naturally D3G-contaminated sample (containing only small amounts of 3ADON) and a D3G-spiked wheat sample were used.

3ADON, 15ADON: Blank wheat samples (0.500 ± 0.002 g) were weighed into an 8-mL vial and spiked with single standard solutions of 3ADON or 15ADON (500 μg kg^−1^) in triplicate. Hydrolysis was performed with 4 mL of 0.1 mM KOH at 60 °C in the laboratory heater for 8 h. The samples were vortexed every 2 h during hydrolysis. Afterwards, 40 μL of 10 mM aqueous HCl were used for neutralization and the samples were cooled down. For extraction, 4 mL of acetonitrile were added to the samples in order to get a final extraction composition of acetonitrile:water 1:1, *v/v*. The samples were extracted on a GFL 3017 rotary shaker (Burgwedel, Germany) for 30 min. The extracts were transferred to HPLC vials and centrifuged on an Awel MF 48-R centrifuge (Blein, France) at 4500 rpm (3830× *g*) prior to injection. Moreover, further concentrations of KOH solution were tested. Therefore, 0.500 ± 0.002 g of a blank wheat sample were weighed into an 8-mL vial and spiked with 3ADON and 15ADON, each present at 500 μg kg^−1^, in triplicate. Hydrolysis was performed with 4 mL of KOH solution (2 mM, 10 mM, or 0.1 M) at 60 °C in the laboratory heater for either 240 or 480 min. The subsequent steps were the same as for the hydrolysis with 0.1 mM KOH.

D3G: The hydrolytic method of Tran and Smith [15] was followed with minor modifications. To decrease the cost of analysis, the whole sample preparation procedure was miniaturized (from 5 g to 0.5 g of sample), but all the conditions used (i.e., temperature, time of hydrolysis, ratios of sample weight/acid volume/extraction solvent volume/neutralization agent volume) remained the same. Blank wheat samples (0.500 ± 0.002 g) were weighed into an 8-mL vial and spiked with 500 μg kg^−1^ D3G in triplicate. Afterwards, 3.6 mL of deionized water were added and the mixture was shaken on the rotary shaker for 30 min. Hydrolysis was performed with 100 μL of 0.5 M TFMSA at 40 °C for 40 min in the laboratory heater. The samples were neutralized with 1 M K_2_CO_3_. The final extracts were transferred to HPLC vials and centrifuged at 4500 rpm (3830× *g*) prior to injection. The same protocol was also applied to the naturally contaminated wheat sample containing 3520 μg kg^−1^ DON, 460 μg kg^−1^ D3G, and 67 μg kg^−1^ 3ADON. In order to verify the stability of DON in the matrix under these conditions, another blank sample was spiked with DON at a level of 3500 μg kg^−1^.

### In-house method validation

Sample preparation for the published indirect methods for the determination of DON and the total amount of DON after acidic hydrolysis with trichloroacetic acid [13], trifluoroacetic acid [14], and trifluoromethanesulfonic acid [15] was performed. Again, only 0.5 g of cereal were used in our case, keeping all other conditions and ratios as specified in the respective publications. Each method was validated for the matrices used in the respective paper, i.e., wheat [13], barley [14], and wheat and maize [15]. In addition, the matrix was validated before hydrolysis (nonhydrolyzed matrix, NHM) and after hydrolysis (hydrolyzed matrix, HM) in order to assure method accuracy.

Two sets of standard stock solutions in acetonitrile were prepared for spiking experiments: (i) individual solutions of DON, D3G, 3ADON, and 15ADON at a concentration of 10 μg mL^−1^ for the HM validation, and (ii) a mixed solution of all toxins used at level of 10 μg mL^−1^ for the NHM validation. In addition, further dilutions were prepared from the 10 μg mL^−1^ mixed solution of all toxins for the preparation of solvent and matrix-matched standards.

Concerning the HM validation, each matrix (0.500 ± 0.002 g) was spiked with single standards of DON, D3G, 3ADON, and 15ADON at 5 μg kg^−1^ in triplicate and stored at room temperature for 2 h. Afterwards, the following method protocols were used. (i) TCA hydrolysis: 0.5 g of spiked sample were extracted with 5 mL of acetonitrile/water (84:16, *v/v*) for 30 min on a rotary shaker. Afterwards, 2 mL of deionized water and 1 mL of 1 M TCA were added. Solvolysis was performed at 140 °C in the laboratory oven for 40 min. After the mixture had cooled down, 0.5 mL of 1 M KOH were added for neutralization and the volume was adjusted to 10 mL with pure acetonitrile. (ii) TFA hydrolysis: 0.5 g of the spiked sample were extracted with 4 mL of acetonitrile/water (84:16, *v/v*) for 30 min on the rotary shaker. Hydrolysis was performed with 100 μL of 1.25 M TFA at 133 °C in the laboratory oven for 54 min. After cooling down, 125 μL of 1 M KOH were added for neutralization. The extract was mixed and transferred into an HPLC vial. (iii) TFMSA hydrolysis: 0.5 g of spiked sample were extracted with 3.6 mL of deionized water and shaken for 30 min on the rotary shaker. Afterwards, 100 μL of 0.5 M TFMSA were added and hydrolyzed for 40 min (wheat) or 20 min (maize) at 60 °C in the lab heater. The whole mixture was adjusted with 1 M sodium bicarbonate to 4 mL.

In addition, the pH was measured four times in each experiment: before acid addition, directly after acid addition, after hydrolysis, and after the addition of the neutralization agent. The pH was measured using a pH electrode on a Microprocessor pH 537 pH meter (WTW, Weilheim, Germany), which was calibrated with buffers of pH 4 and 7 (for acidic conditions) or buffers of pH 7 and 10 (for alkaline conditions).

Similarly, the NHM validation was performed using the same protocol as used for the HM validation but with two modifications. Firstly, samples were spiked with the mixed solution of all toxins. Secondly, water was used instead of acid in the protocol described above, and no KOH was added for neutralization.

Two types of calibration curves covering the concentration range 16.6–750 μg L^−1^ were prepared. Appropriate amounts of the mixed solution were evaporated under a gentle nitrogen stream and the standards were re-dissolved in 1 mL of acetonitrile/water 84:16, *v/v* for the solvent calibration curve. Similarly, matrix-matched calibrations for NHM and HM were prepared by re-dissolving dried-down standards in 1-mL blank extracts from the respective matrices.

Method performance characteristics were calculated according to the following equations:

- Apparent recovery (*R*_A_):
  1 $$ {R}_{\mathrm{A}}\left(\%\right)=\frac{\mathrm{slope}\ \left(\mathrm{spiked}\ \mathrm{samples}\right)}{\mathrm{slope}\ \left(\mathrm{neat}\ \mathrm{solvent}\ \mathrm{standard}\right)}\times 100. $$
- Matrix effect as a signal suppression/enhancement (SSE):
  2 $$ \mathrm{S}\mathrm{S}\mathrm{E}\ \left(\%\right)=\frac{\mathrm{slope}\ \left(\mathrm{matrix}\mathrm{\hbox{-} matched}\ \mathrm{standard}\right)}{\mathrm{slope}\ \left(\mathrm{neat}\ \mathrm{solvent}\ \mathrm{standard}\right)}\times 100. $$
- Extraction recovery (*R*_E_):
  3 $$ {R}_{\mathrm{E}}\left(\%\right)=\frac{R_{\mathrm{A}}}{\mathrm{SSE}}\times 100. $$

DON was quantified after the hydrolysis of samples spiked with D3G, 3ADON, or 15ADON using the HM-matched calibration curve. The percentage of DON released was calculated as follows:

4 $$ \mathrm{DON}\ \mathrm{release}\ \left(\%\right)=\frac{\mathrm{quantified}\ \mathrm{DON}\ \mathrm{level}}{\mathrm{spiked}\  \mod .\mathrm{toxin}\ \mathrm{level}}\times \frac{M\left( \mod .\mathrm{toxin}\right)}{M\left(\mathrm{DON}\right)}\times 100M=\mathrm{molar}\ \mathrm{mass}\left({\mathrm{g}\ \mathrm{mol}}^{-1}\right). $$

### UHPLC-MS/MS analysis

A 1290 series UHPLC system (Agilent Technologies, Waldbronn, Germany) coupled to a QTrap 5500 MS/MS system from AB Sciex (Foster City, CA, USA) was used for analysis. Chromatographic separation was performed on an Acquity UPLC BEH column (50 × 2.1 mm, 1.7 μm, Waters, Milford, MA, USA) held at 40 °C. Water/acetic acid (99:1, *v/v*) was used as eluent A and eluent B consisted of methanol/water/acetic acid 95:4:1, *v/v/v*. Both eluents also contained 5 mM ammonium acetate. Gradient elution started at 10 % B, which slowly increased to 20 % B after 2 min, and then increased linearly to 40 % B in another 4 min. 90 % B was reached by performing a rapid increase for another minute. Re-equilibration for another 2 min at 10 % B was realized prior to further injection. The flow rate was 500 μL min^−1^ and the injection volume was 2 μL.

The QTrap 5500 was operated in electrospray ionization mode using a TurboV ion spray source with the following settings: curtain gas (CUR), 30 psi (207 kPa, nitrogen); collision gas (CAD, nitrogen), medium; ion spray voltage, −4500 V/+4500 V; temperature, 550 °C; sheath gas (GS1) and drying gas (GS2), both 80 psi (552 kPa, zero-grade air). Acquisition was performed in the selected reaction monitoring mode and the chromatographic run was divided into two individual periods with a settling time of 50 ms. The mass spectrometric conditions for the individual analytes are provided in Table 1.

**Table 1:** Optimized tandem mass spectrometric conditions for deoxynivalenol (DON), DON-3-glucoside (D3G), 3-acetyl-DON (3ADON), and 15-acetyl-DON (15ADON)

Analyte	Retention time (min)	Period/ion	Precursor ion (*m/z*)	Product ions (*m/z*) ^a^	DP (V)	CE (V)	CXP (V)	Dwell time (ms)
DON	1.20	1/ [M + CH_3_COO]^−^	355.1	58.9	−60	−52	−7	30
				295.1	−60	−14	−11	30
				265.1	−60	−22	−9	30
D3G	1.35	1/ [M + CH_3_COO]^−^	517.1	457.1	−80	−18	−17	30
				427.1	−80	−28	−15	30
3ADON	3.33	2/ [M + H]^+^	339.1	203.0	116	19	8	30
				231.0	116	17	18	30
				213.0	116	19	10	30
15ADON	3.45	2/ [M + H]^+^	339.1	321.0	126	13	14	30
				137.0	126	15	8	30
				261.0	126	17	12	30

*DP* declustering potential, *CE* collision energy, *CXP* collision cell exit potential

^a^The first product ion was used as quantifier, the other two transitions served as qualifiers

## Results and discussion

### Hydrolysis experiments with pure standards

The first criterion of an indirect method is that the final product (the compound which is released during the reaction and determined afterwards) has to be stable during the chosen hydrolytic conditions. Therefore, suitable conditions for hydrolysis must be found which leave DON intact. The reagents and conditions of the experiments on the pure standards were based on those used in previous reports [13–15, 19].

When KOH was used in the hydrolysis of acetylated conjugates of DON, it had no impact on DON stability at concentrations of 1 μM (pH 8) and 0.1 mM (pH 10) up to 100 °C for 8 h. Higher concentrations resulted in detectable DON degradation after 1 h. Higher temperatures resulted in even faster degradation. TCA caused fast DON degradation at each tested concentration. The conditions employed by Liu et al. [13], 1 M TCA at 130 °C, led to DON losses of almost 50 % after 30 min. 0.1 M and 0.2 M TFMSA had no impact on the stability of DON at temperatures of up to 60 °C. The conditions used by Tran and Smith [15], 0.5 M TFMSA at 37 °C for 4 h, seemed to be suitable, as losses of DON did not exceed 5 %. However, fast DON degradation was observed at temperatures exceeding 40 °C.

The conditions which passed the first criterion (no impact on DON stability) were used for the hydrolysis of individual standards of D3G, 3ADON, and 15ADON. As expected, D3G was stable at 0.1 mM KOH at any temperature and duration tested. Using 0.1 M TFMSA for the hydrolysis of D3G was also inefficient. Increasing the concentration of TFMSA to 0.5 M caused the rapid decomposition of D3G to unknown products at 37 °C and no release of DON was observed. Slow degradation of 3ADON was observed when 0.1 M TFMSA was used. The loss reached 11–23 % depending on the temperature applied (the higher the temperature, the greater the loss). In contrast, 0.1 M TFMSA had no effect on 15ADON regardless of the temperature used and time allowed. Hydrolysis of both 3ADON and 15ADON to DON was achieved in 1 mM KOH at 60 °C for 8 h. The hydrolysis rates for 3ADON and 15ADON were 90 and 95 %, respectively. There was gradual degradation of the DON formed at longer reaction times. Therefore, the following conditions were used as initial conditions for the subsequent experiments on the wheat matrix: 1 mM KOH, 60 °C, 8 h.

### Alkaline hydrolysis of conjugated DON in the wheat matrix

Using 1 mM KOH at 60 °C for 8 h to hydrolyze 3ADON and 15ADON spiked into wheat did not prove to be successful. On the one hand, 3ADON and 15ADON were not completely hydrolyzed to DON, as only 32 % of the spiked 3ADON and 47 % of the spiked 15ADON were hydrolyzed. On the other, the levels of DON released were lower than expected (less than 25 %) and did not correspond to the amounts of hydrolyzed 3ADON and 15ADON. Moreover, it was revealed that the matrix buffers the pH of the hydrolytic agent from pH 10 to pH 6. Therefore, the concentration of KOH was increased and the experiment was repeated. The results are summarized in Table 2. Interestingly, the hydrolysis rates contradict those obtained in the first matrix experiment with 1 mM KOH. Increasing the concentration of KOH had only a small impact on the hydrolysis of acetylated DONs. For instance, using 2 mM KOH at 60 °C for 8 h hydrolyzed only 6 % of both 3ADON and 15ADON. Fast degradation of 3ADON without further DON release was observed with the highest KOH concentration tested (0.1 M). Furthermore, the molar sum of 3ADON, 15ADON, and DON was higher than the initial sum of these toxins before hydrolysis. This may have been caused either by the presence of additional (unknown) sources of DON in the sample or signal enhancement in LC-MS/MS.

**Table 2:** Release of deoxynivalenol (DON) and residual recoveries of 3-acetyl-DON (3ADON) and 15-acetyl-DON (15ADON) after alkaline hydrolysis of a wheat blank sample spiked at 500 μg kg^−1^ with 3ADON and 15ADON

Analyte (%)	Hydrolytic conditions					
	2 mM KOH, 60 °C, 4 h	2 mM KOH, 60 °C, 8 h	10 mM KOH, 60 °C, 4 h	10 mM KOH, 60 °C, 8 h	0.1 M KOH, 60 °C, 4 h	0.1 M KOH, 60 °C, 8 h
DON	43	50	49	52	54	40
3ADON	91	94	92	88	4	4
15ADON	97	94	94	93	79	85

### Acidic hydrolysis of D3G in the wheat matrix

As no suitable conditions for the alkaline hydrolysis of D3G were found, the method of Tran and Smith using TFMSA [15] was applied to both spiked wheat samples and naturally contaminated wheat. Although D3G disappeared, no DON increase was observed. The reaction seemed to lead to the formation of other products. Interestingly, D3G degradation occurred to a lesser extent in the naturally contaminated sample (55 % of D3G initial levels detected after hydrolysis) compared to the spiked one where only 13 % of D3G were recovered. D3G seems to be better protected by the matrix in naturally contaminated samples. A negative control sample (sample spiked only with DON) was prepared in order to verify the stability of DON. The recovery of DON after hydrolysis was very low, yielding only 31 % of the expected amount. There are three theoretical reasons for this phenomenon: (i) DON is unstable during hydrolysis—which can be excluded based on the results reported above; (ii) low extraction efficiency of DON from the hydrolyzed matrix; or (iii) matrix effects in LC-MS/MS.

### In-house method validation

As we did not find any reports of suitable conditions for acidic or alkaline hydrolyses of either acetylated DONs or D3G, our doubts over the effectiveness of previously published indirect methods for total DON determination [13–15] grew. In order to avoid the generation of biased data due to matrix effects, the developed LC-MS/MS method had to be properly validated. Therefore, sample preparation procedures used in published methods [13–15] were combined with the newly developed LC-MS/MS method and validated. The matrix was visibly changed after hydrolysis, so matrix effects must have influenced DON determination in the matrix before and after hydrolysis in different ways. Moreover, it was also suspected that the extraction recovery of DON from NHM was different from the extraction recovery of DON from HM. In order to compare the method accuracy for free (extractable) DON with the accuracy for total DON determination, we decided to validate both matrices (NHM and HM). The performance characteristics of the methods are summarized in Table 3.

**Table 3:** Validation data for the indirect approaches to DON determination using UHPLC-MS/MS

Agent (matrix)	Declared increase in DON after hydrolysis (%)	Analyte	Nonhydrolyzed matrix (NHM)				Hydrolyzed matrix (HM)*				DON released (%, *n* = 3)
			*R*_E_ (%)	*R*_A_ (%)	SSE (%)	RSD (%)	*R*_E_ (%)	*R*_A_ (%)	SSE (%)	RSD (%)	
TCA (wheat)	13–63 published in [13]	DON	59	63	107	5	74	39	53	6	–
		D3G	82	46	56	1	31	20	65	4	n.d.
		3ADON	62	62	100	2	66	65	98	4	n.d.
		15ADON	60	61	102	3	67	61	91	1	n.d.
TFA (barley)	9–88 published in [14]	DON	101	97	96	2	120	67	56	21	–
		D3G	93	62	67	2	83	41	49	2	n.d.
		3ADON	100	100	100	4	119	111	93	1	n.d.
		15ADON	101	94	93	7	92	96	104	3	n.d.
TFMSA (wheat)	7–75 published in [15]	DON	94	126	134	19	55	82	149	6	–
		D3G	75	150	200	22	11	12	108	1	n.d.
		3ADON	80	103	128	6	86	45	52	9	33
		15ADON	82	76	93	6	47	8	17	10	40
TFMSA (maize)	8–70 published in [15]	DON	103	66	64	4	31	40	128	5	–
		D3G	97	70	72	4	75	54	72	4	n.d.
		3ADON	81	78	96	7	4	2	52	1	31
		15ADON	91	89	98	5	38	5	13	16	33

*R*_*E*_ extraction recovery, *R*_*A*_ apparent recovery, *SSE* signal suppression or enhancement, *RSD* relative standard deviation (repeatability, *n* = 3, calculated from apparent recovery), *DON* deoxynivalenol, *D3G* DON-3-glucoside, *3ADON* 3-acetyl-DON, *15ADON* 15-acetyl-DON, *TCA* trichloroacetic acid, *TFA* trifluoroacetic acid, *TFMSA* trifluoromethanesulfonic acid, *n.d.* not detected, *except in the TFA hydrolysis procedure, the pH was not neutral after the addition of alkaline solution to the HM

Upon comparing the validation data for DON in NHM and HM, huge differences were observed in SSE and* R*_E_ values. Strong suppression of the DON signal in TCA-hydrolyzed wheat matrix and TFA-hydrolyzed barley matrix was observed compared to that seen in the respective NHM. In contrast, TFMSA hydrolysis of wheat and maize resulted in high DON signal enhancement when determined by LC-MS/MS. Although other analytical techniques (GC-ECD, GC-MS, and ELISA) were used as analytical tools in the previously published studies, matrix effects should have been taken into account during method development. GC-based methods, just like LC-MS/MS methods, can suffer from matrix effects or other difficulties which hinder accurate determination. It has been stated that GC-based techniques in particular (such as GC-ECD, GC–flame ionization detection, or GC-MS), when used for trichothecene determination, are prone to signal enhancement even when clean-up is performed [20, 21]. Moreover, aside from signal enhancement (the overestimation of a toxin due to matrix effects), other problems such as nonlinearity of calibration curves, poor repeatability, or memory effects from a previous sample injection [20, 21] could have led to the overestimation of DON in HM that was observed in the previously published studies [13, 14]. Further, the analytical methods used in these studies were not properly described. There is no information about method linearity and repeatability. More importantly, it is not clear whether the results were calculated using solvent- or matrix-matched calibration curves. In addition, the extraction recovery of DON is higher after TCA and TFA hydrolysis than from NHM. Thus, the final free DON and total DON levels should have been corrected for recovery.

The indirect approach reported by Tran and Smith in 2011 [15] was verified using an AgraQuant^®^ DON assay 0.25/5.0 test kit (Romer Labs Inc., Union, MO, USA). All samples were analyzed for free DON, 3ADON, and 15ADON by GC-MS, but D3G was not taken into account. After hydrolysis, no confirmation of the accuracy of the ELISA results was performed by GC-MS. The authors of this study nevertheless claimed that the increase in DON levels after hydrolysis was caused by the release of this toxin from masked forms other than 3ADON or 15ADON that were either present at low levels or below the limit of quantification. It was furthermore argued that D3G could not have contributed to the “total” DON level because it is unstable under the applied hydrolytic conditions. In general, antibody-based methods are considered to be useful tools for screening purposes, but the quantitative results obtained should be verified using a “confirmatory method” [22]. The main risk is of overestimating the results due to antibody cross-reactivity, which was most likely observed by the authors, rather than the release of DON. The results obtained in this study are doubtful for several reasons. First of all, AgraQuant^®^ was found to be a kit that overestimates the DON content compared to LC-MS/MS results [23–25]. Two comprehensive studies performed on four commercially available DON ELISA kits revealed that DON overestimation is caused by both cross-reactivity with structurally related compounds and by matrix effects. Although the kit manufacturer stated that the cross-reactivity with 3ADON was >100 %, two studies reported it to be more than 300 % based on the solvent standard [24, 25]. Cross-reactivity with D3G was found to be 113 % in the latter study [25], while Tran and Smith [15] based their calculations on a mere 4.8 %, as specified by the manufacturer. Furthermore, a matrix contribution to DON overestimation was reported [24] several years before the published hydrolytic method of Tran and Smith [15]. A certified reference material of wheat was subsequently analyzed by LC-MS/MS, ELISA, and ELISA after MycoSep^™^ 226 clean-up [24]. The results obtained using the latter approach corresponded to those determined by LC-MS/MS, while analysis of the raw extract by ELISA resulted in a huge overestimate of the DON in addition to the effect caused by cross-reactivity with 3ADON, 15ADON, and D3G [24]. It is likely that the increase in DON levels after hydrolysis, considered to be due to the release of masked forms of this toxin, were actually caused by a combination of matrix effects (cross-reactivity of matrix co-extracts), the huge cross-reactivity of 3ADON, and the cross-reactivity of degraded D3G. In general, it is recommended that matrix-matched calibration curves should be used in ELISA analysis in order to decrease overestimation. It has been proven that every matrix behaves differently in terms of the cross-reactivity caused by matrix co-extracts [24, 25]. Based on our LC-MS/MS validation data, this is also true of nonhydrolyzed and hydrolyzed versions of the same matrix. Although the authors’ conclusion [15] that D3G is unstable under TFMSA hydrolysis has been confirmed, D3G may still have contributed to the total DON result. The unknown degradation products of D3G formed during hydrolysis may be cross-reactive to some extent.

Our doubts that none of the published indirect methods work properly were also confirmed by the fact that DON was not detected after TCA and TFA hydrolyses in the samples spiked with pure 3ADON, 15ADON, and D3G standards. This means that these compounds—if they were degraded at all—were not cleaved to DON. In addition, the stability of 3ADON and 15ADON towards acidic hydrolysis is supported by validation data. There are no differences in* R*_E_ and SSE for both acetylated forms of DON when comparing NHM with HM. However, in terms of the validation data for 3ADON and 15ADON in TFMSA hydrolysis, unexpectedly low values of* R*_E_,* R*_A_, and SSE were obtained in HM (Table 3). Moreover, released DON was detected in 3ADON- and 15ADON-spiked samples after the hydrolysis procedure. The initial experiments with pure standards revealed that 3ADON and 15ADON are fully stable under the acidic conditions used, but they can be cleaved under alkaline conditions. In order to explain those results, the next step was to perform a small-scale hydrolysis experiment and to measure the pH at each step of the procedure. The results are summarized in Table 4. Thus, in two of the three studies, 3ADON and 15ADON were most likely not cleaved during the acidic hydrolysis with TCA or TFMSA, but they were after the addition of an excess of the neutralization agent. The reason that this phenomenon was only observed in the validation data for TFMSA hydrolysis was the order of measurement of the samples. Validation samples of TFMSA hydrolysis were the last in the queue to be measured, >8 h after sample preparation (“neutralization”). Also, the matrix-matched standards of 3ADON and 15ADON prepared using blank extracts of hydrolyzed and “neutralized” matrices slowly decomposed during analysis due to the alkaline conditions present.

**Table 4:** pH values obtained during indirect methods for total deoxynivalenol determination

Matrix	Before hydrolysis	After acid addition	After hydrolysis	After neutralization
Wheat (TCA)	6.18	1.16	1.61	13.22
Barley (TFA)	6.24	1.70	2.16	6.93
Wheat (TFMSA)	5.86	3.37	3.52	10.08
Maize (TFMSA)	6.11	3.15	3.97	10.07

*TCA* trichloroacetic acid, *TFA* trifluoroacetic acid, *TFMSA* trifluoromethanesulfonic acid

## Conclusions

A critical assessment of three indirect methods for total DON determination based on acidic hydrolysis using TCA [13], TFA [14], or TFMSA [15] was carried out in this study. The first phase of the study focused on the stability/degradation of pure standards of DON, D3G, 3ADON, and 15ADON under various hydrolytic conditions, which was followed by experiments on a wheat matrix. As none of the hydrolytic conditions were found to be suitable for achieving the reliable decomposition of modified forms of DON to the parent toxin (DON), we decided to assess protocols for previously published indirect methods. The most important findings were:

- Validating both the method for “free DON” (NHM) and the method for “total DON” (HM) revealed huge differences in SSE as well as in* R*_E_ when DON was determined in NHM and HM. The authors of previous studies [13–15] did not take into account the changes caused to the matrix by hydrolysis. As no information on method performance characteristics and data evaluation or any corrections to the results (in terms of recovery and matrix effects) was provided, the observed DON increase was most likely caused by DON signal enhancement during HM analysis, signal suppression during NHM analysis, or both.
- The exclusion of D3G—the major masked form of DON—as a model toxin in indirect method development in previous studies [13–15] renders these methods unsuitable for routine use.
- An intended neutralization step using aqueous KOH or K_2_CO_3_ after hydrolysis led to alkalinization of the cereal extracts. Under those conditions, 3ADON and 15ADON (and potentially other modified forms) were cleaved to DON and thus further increased the level of DON (or further breakdown products). The increase in DON was not caused by acidic hydrolysis, as claimed by the authors.

In summary, the use of acidic or alkaline hydrolytic procedures for the indirect determination of total DON is strongly discouraged. Indirect methods using enzymatic hydrolysis show more promise. A 1,3-β-glucanase with high affinity for D3G was identified, but this enzyme was strongly inhibited under the matrix conditions employed here, and sample clean-up was required before enzymatic hydrolysis [26]. Recently, a highly efficient glucosidase from *Bifidobacterium* that is able to hydrolyze D3G in the matrix was identified [27]. Nevertheless, direct LC-MS/MS utilizing standards for known modified forms presently remains the method of choice to determine the levels of DON and its modified forms in cereals and cereal-based food.
